# Phylogenetic and recombination analysis of human bocavirus 2

**DOI:** 10.1186/1471-2334-11-50

**Published:** 2011-02-24

**Authors:** Weixia Cheng, Jinan Chen, Ziqian Xu, Jiemei Yu, Canping Huang, Miao Jin, Huiying Li, Ming Zhang, Yu Jin, Zhao-jun Duan

**Affiliations:** 1 State Key Laboratory for Molecular Virology and Genetic Engineering, National Institute for Viral Disease Control and Prevention, China CDC, Beijing 100052, PR China; 2 Medical School of Nanjing University, Nanjing Children's Hospital, Nanjing 210093, PR China; 3 School of Basic Medical Sciences, Lanzhou University, Lanzhou 730000, PR China; 4 National Laboratory of Biomacromolecules, Institute of Biophysics, Chinese Academy of Sciences, Beijing 100101, PR China; 5 Dept. of Epidemiology and Biostatistics and Faculty of Infectious Diseases, University of Georgia, Athens, GA 30602 USA

## Abstract

**Background:**

Human bocavirus 2(HBoV2) and other human bocavirus species (HBoV, HBoV3, and HBoV4) have been discovered recently. But the precise phylogenetic relationships among these viruses are not clear yet.

**Methods:**

We collected 632 diarrhea and 162 healthy children in Lanzhou, China. Using PCR, Human bocavirus (HBoV), HBoV2, HBoV3 and HBoV4 were screened. The partial genes of *NS*, *NP1 *and *VP*, and two nearly complete sequences of HBoV2 were obtained.

**Result:**

Phylogenetic analysis showed the different genes of HBoV2 strain were homogenous with different reference strains. HBoV3 may be a recombinant derived from HBoV and HBoV4. We also observed that the VP1 and VP2 region of HBoV3 is as similar to HBoV2 as to HBoV4.

**Conclusions:**

A single genetic lineage of HBoV2 is circulating in children with and without gastroenteritis in Lanzhou, China. Current evidence in this study was not enough to support recombination between HBoV2 strains, and HBoV3 may be a recombinant between HBoV and the common ancestor of HBoV2 and HBoV4.

## Background

Human bocavirus (HBoV), HBoV2, HBoV3, and HBoV4 have been discovered recently [1-4]. These viruses belong to the genus *Bocavirus *in the subfamily *Parvovirinae *of the family *Parvoridae*, among which the human parvovirus B19 (B19V) is the only known human pathogen [5]. HBoV was detected in respiratory tract samples in 2005[1]. In 2009, Kapoor *et al. *[2] reported a new bocavirus species, HBoV2, isolated from stool samples in children with nonpolio acute flaccid paralysis, and suggested that recombination between HBoV2 strains may occurs. Almost simultaneously, Arthur *et al. *[3] reported that HBoV3 was detected in stool samples from children with acute gastroenteritis (AGE). In addition, they proposed that HBoV3 is a hybrid of HBoV and HBoV2. Subsequently these two new viruses were detected in nasopharyngeal aspirates or stool samples in other regions [6-11]. Recently Kapoor *et al *reported the discovery of HBoV4 and the detection of recombination signals between and within bocavirus species [4]. Among these human bocaviruses, HBoV2 had higher prevalence and genetic diversity in stool samples than the others [3,8], but the precise phylogenetic relationships among these viruses were not clear yet. Furthermore, the prevalence and genetic feature of HBoV2 were not addressed at children with and without AGE.

In the present study we collected stool samples from children with and without AGE in Lanzhou, China. Samples were assayed for the presence of HBoV, HBoV2, HBoV3 and HBoV4. Partial nucleotide sequences of the *NS*, *NP1*, *VP1/2 *genes, and two nearly full-length genome sequences of HBoV2 were obtained.

## Methods

### Ethics statement

The study was approved for human subject protection by the Research Ethics Committee of the Lanzhou University and the Institutional Review Board at China CDC. Following informed consent was written by parent/guardian.

### Patients and methods

From July 2006 to June 2008 in Lanzhou, China, we collected stool samples from 632 hospitalized children with diarrhea and 162 asymptomatic children. 3-5 ml stool was collected from every participant. All subjects were 5 or less years of age. Medical histories were provided by parents/guardians. The case group included subjects hospitalized for gastroenteritis in the Department of Pediatrics in our institution. Diarrhea was defined as three or more loose stools in the previous 24 h. Patients were excluded from the study if stool sample volume was insufficient for a complete evaluation of viral agents, stools had blood streaks or pus, or due to the presence of a co-morbidity. Subjects in the control group had presented to the First Hospital of Lanzhou University Pediatric Primary Care Center for a routine examination and did not have fever, diarrhea, vomiting, or respiratory illness in the previous 3-week period [12]. Control subjects received follow-up by telephone and those in whom any of the aforementioned exclusion criteria were present during the week after the initial examination were excluded. A 10% suspension of stool sample was made by mixing 0.5 g stool with 1.0 mL PBS (pH7.2). Viral RNA and DNA were extracted from stool suspensions clarified by centrifugation (1500 × *g*, 20 min) using a QIAamp^® ^Viral RNA Mini kit (Qiagen, Hilden, Germany), according to the manufacturer's protocol. RNA and DNA were resuspended in 50 μL water and stored at -70°C.

### Detection of HBoV, HBoV2, HBoV3, and HBoV4

HBoV was detected using a method described by us previously [13]. HBoV2, HBoV3, and HBoV4 were detected by nested PCR as described by Kapoor *et al*., in which primers HBoV2-sf1, HBoV2-sr1, HBoV2-sf2, and HBoV2-sr2 were used to amplify a 495-nt region within the ORF of NS1 [2]. PCR-positive samples were confirmed by sequencing.

### Sequence and phylogenetic analysis

To analyze genetic variation in HBoV2, two sets of nested primers were designed and used to amplify part of the *NP1 *and *VP1 *genes in positive samples (Table 1), using the NP1-F1 and NP1-R1, and VP1-F1 and VP1-R1 primers in the first round of PCR and the NP1-F2 and NP1-R2, and VP1-F2 and VP1-R2 in the second. The reaction mix contained 10 pmol each primer and 2.5 units ExTaq DNA polymerase (Takara Bio). After 5 min at 94°C, 35 cycles of amplification (94°C for 45 s, 50°C for 1 min, and 72°C for 1 min) were performed, followed by a 7 min extension at 72°C. Complete HBoV2 sequences were amplified by using specific PCR and Genome Walking Kit (TaKaRa code: D316). PCR products were cloned and the plasmid inserts were sequenced. Nucleotide and deduced amino acid sequences were compared to entries in the GenBank database. Phylogenetic analysis was conducted with Molecular Evolutionary Genetics Analysis (MEGA) version 4.1. All *NS1*, *NP1*, *VP1 *partial gene sequences and nearly full-length genome sequences of HBoV2 except the termini were submitted to GenBank (accession numbers GU301644-GU301683, HQ153797-HQ153804).

**Table 1:** Primers used in this study.

Primer	Sequence	Target
NP-F1(+)	ATACGTGGCAGTCACAACCT	*NP1*
NP-R1(-)	CGTCTGTTAC CTCCTCTGAT	*NP1*
NP-F2(+)	ATGAGCTCCGAATCTATG	NP1*
NP-R2(-)	CCTCTGATTCCTGTGAAG	*NP1*
VP-F1(+)	AACGACTGGTCTCTTGGTGGCAT	*VP1*
VP-R1(-)	TGGATATGCG TGTTCACCAT CAC	*VP1*
VP-F2(+)	CGACTGGTCTSTTGGTGGCATTAT	*VP1^#^*
VP-R2(-)	CGTTGTTGTA TGTAGTGTCA GCACC	*VP1*

* The target fragment of *NP1 *partial gene was 586 nt, in 2285-2870 nt according to HBoV2 FJ170279 strain.

^# ^The target fragment of *VP1 *partial gene was 620 nt, in 3134-3753 nt according to HBoV2 FJ170279 strain.

^‡^The target fragment of NP1 Real-time PCR 145 nt, in 2489-2633 nt according to HBoV2 FJ170279 strain.

A total of 82 nearly full-length genome sequences of HBoV, HBoV2, HBoV3, and HBoV4 were obtained from GenBank. These were aligned and manually adjusted using ClustalW and BioEdit. Phylogenetic trees were determined by the neighbor-joining (NJ) method using the MEGA 4.1 software package. Various nucleotide substitution models were examined and yielded phylogenetic trees of similar topology; only the Kimura 2-parameter model trees were described in this report. A bootstrap resampling (1000 replications) was used to assess the reliability of individual nodes in each phylogenetic tree.

### Recombination Analyses

We detected recombination using the RDP3 package. Sequences were selected based on their similarity and then aligned and manually adjusted using ClustalW and BioEdit. The alignments were scanned by various algorithms implemented in the RDP3 package, followed by manual refinement. In addition, we performed similarity plot and bootscanning analyses for potential recombination events using SimPlot 3.5. Genetic Algorithm Recombination Detection (GARD) methods were also used to detect recombination and estimate breakpoint locations. Estimated breakpoints were verified by both the Shimodaira-Hasegawa test and manually checking phylogenetic trees for nonrecombinant segments.

## Results

### Detection of human bocaviruses

In subjects with gastroenteritis, the positive rates of HBoV, HBoV2 and HBoV3 were 4.3%, 20.4% and 0.9%, respectively, whilst HBoV4 was not detected. In the control group, HBoV and HBoV2 were detected in 2.5% and 12.3% of subjects; HBoV3 and HBoV4 were not detected.

### Phylogenetic analyses of HBoV2

**Figure 1:**
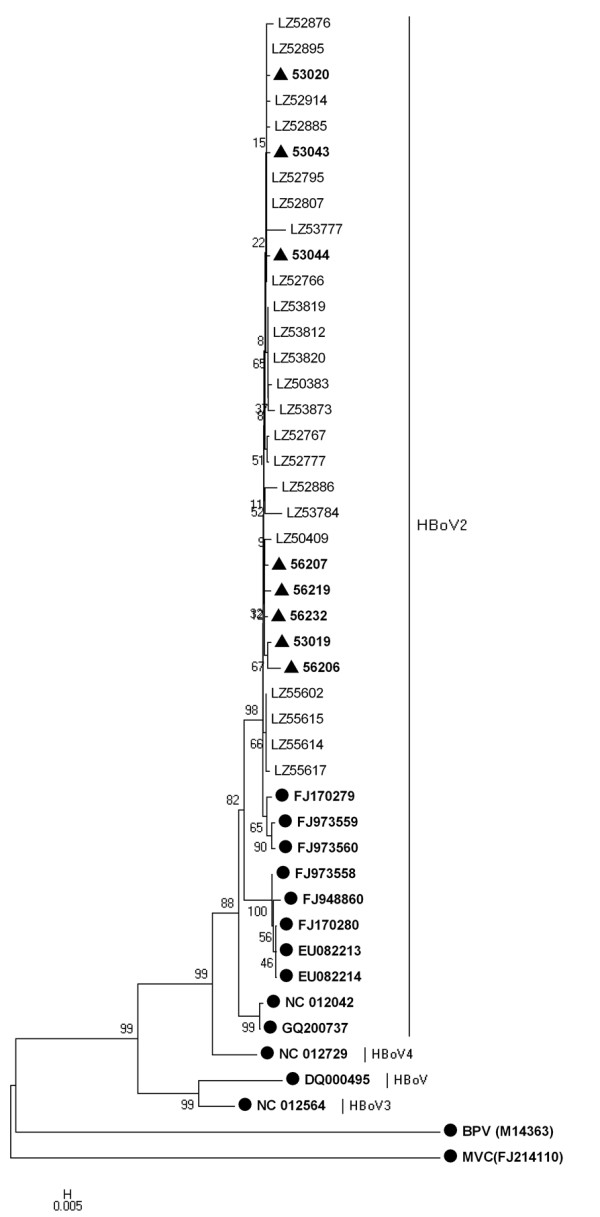
**Phylogenetic analysis of human bocavirus1-4 and other bocavirus members partial *NS1 *gene sequences**. Phylogenetic tree was constructed by the neighbor-joining method, Kimura 2-parameter model with 1,000 bootstrap replicates, by using MEGA 4.1 package. Black dots designate reference strains, black triangles designate sequences from control group and the others were sequences generated from the gastroenteritis children in the present study. MVC: Minute virus of canines; BPV: Bovine parvovirus.

Phylogenetic analysis indicated that all of the partial *NS1 *gene sequences of case group were closer with the PK-2255 strain (from Pakistani children, GenBank: FJ170279) than other strains, with 97.4-99% identity. Consistently, those of control group had 98-99% identity with the PK-2255 strain and were also closer with it than others. The sequences in this study, including case and control group, had a high identity of 98.5-100% with each other. The Phylogenetic tree also indicated that there was no difference in the topological characteristics of HBoV2 between case and control groups (Figure 1).

**Figure 2:**
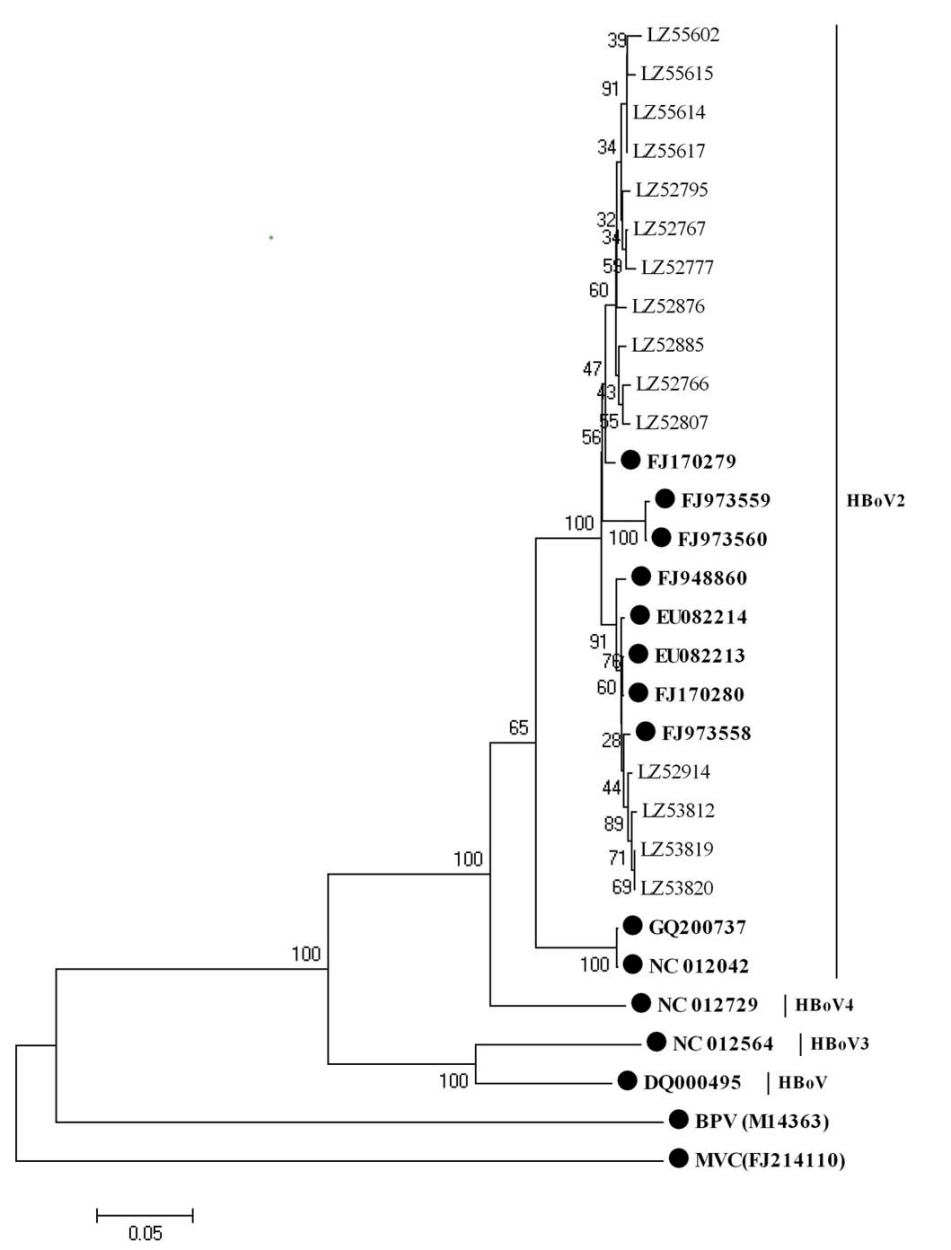
**Phylogenetic analysis of the partial *NP1 *gene sequences of human bocavirus1-4 and other bocavirus members**. Phylogenetic tree was constructed by the neighbor-joining method, Kimura 2-parameter model with 1,000 bootstrap replicates, by using MEGA 4.1 package. Black dots designate reference strains, the others were sequences generated from the present study. MVC: Minute virus of canines; BPV: Bovine parvovirus.

**Figure 3:**
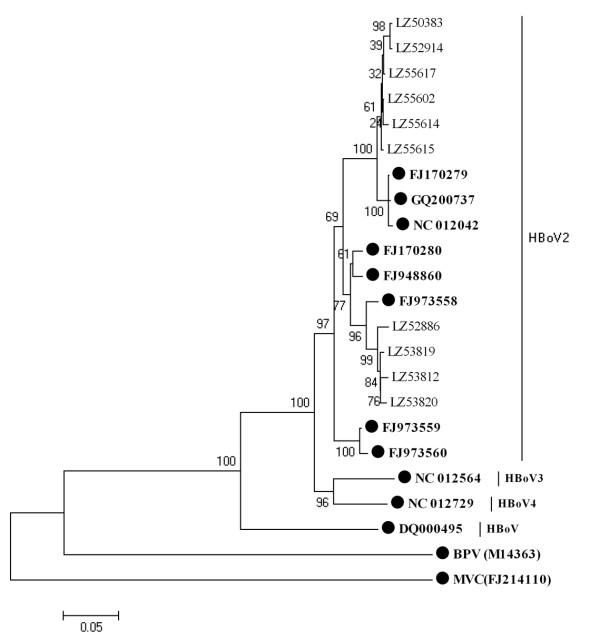
**Phylogenetic analysis of the partial *VP1 *gene sequences of human bocavirus1-4 and other bocavirus members**. Phylogenetic tree was constructed by the neighbor-joining method, Kimura 2-parameter model with 1,000 bootstrap replicates, by using MEGA 4.1 package. Black dots designate reference strains, the others were sequences generated from the present study. MVC: Minute virus of canines; BPV: Bovine parvovirus.

Further study indicated that partial *NP1 *gene sequences were also similar to those of strain PK-2255 (98-99%), except that four had a high identity to strain FJ973558 (Figure 2), with >99% sequence identity. Interestingly, the ten HBoV2 partial *VP1 *gene sequences in this study were more variable than those of *NS1 *and *NP1*, being only 92.6-97.3% similar to those of strain PK-2255. Six partial *VP1 *gene sequences (Figure 3) were in the same cluster as strain PK-2255 (97.3-98.1% identity) and the other four sequences clustered with strain FJ973558 (96.9-97.4% identity). The nearly full-length genome sequence of LZ53819 generated in this study (GenBank number: GU301644) was more similar to strain FJ973558 than to PK-2255 (similarity 97.5%), but the *NS1 *gene sequence showed greater similarity to that of strain PK-2255. Above results suggested that recombination among HBoV2 genotypes may occur. While we compared the trees of non-recombinant segments associated with the break points estimated by GARD, Shimodaira-Hasegawa test showed the topologies of these trees were not significantly different.

### Recombination analyses among HBoV, HBoV2, HBoV3, and HBoV4

**Figure 4:**
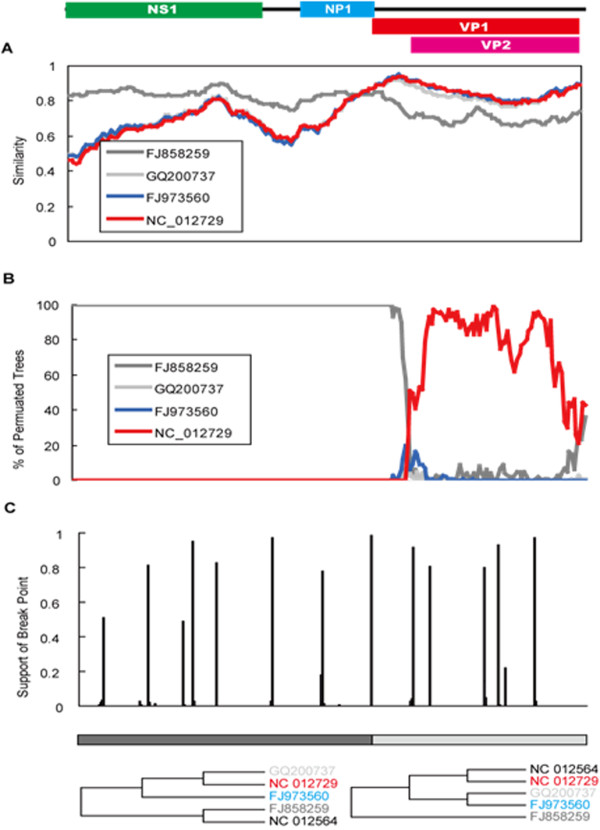
**HBoV3 is a potential hybrid of HBoV and HBoV4 by recombination analysis**. (A) recombination analysis was conducted by Similarity plot, (B) recombination of HBoV3 was conducted by bootscanning analysis, (C) recombination of HBoV3 was conducted by GARD analysis.

The phylogenetic relationships of a total of 82 nearly full-length genome sequences obtained both from this study and GenBank were inferred. Based on sequence identity, we selected alignments of five nearly full-length genome sequences. Recombinant signals were detected in all of the HBoV, HBoV2, HBoV3 and HBoV4 genome sequences, but the signals in HBoV3 was far more significant than those in other bocaviruses. Using RDP3 and SimPlot3.5, it was found that HBoV3 (NC_012564) was a potential recombinant of HBoV (FJ858259) and HBoV4 (NC_012729) (Figure 4A, B). The breakpoint was located near the *VP1 *start codon. GARD analyses suggested six possible breakpoints with model average support over 0.90, including one located 18 bp downstream of the *VP1 *start codon (Figure 4C). This breakpoint was further examined by constructing phylogenetic trees of the two nonrecombinant segments (Figure 4C). While NC_012564 grouped with FJ858259 in the 5' segment (including *NS1 *and *NP1*), it grouped with NC_012729 in the 3' segment (consisting of *VP1 *and *VP2*), suggesting that HBoV3 is a hybrid of HBoV and HBoV4. However, the *VP1 *and *VP2 *region of HBoV3 was as similar to HBoV2 (identity 87.0% with GQ200737 and 88.6% with FJ973560) as it was to HBoV4 (88.3% to NC_012729).

## Discussion

Although the primers used in this study were capable of detecting HBoV4, no HBoV4 was detected. HBoV2 had higher prevalence than HBoV and HBoV3 in both case and control groups, indicating that HBoV2 should be given more attention than other human bocaviruses. Given the fact that children in the control group that were free of symptoms were positive for HBoV and HBoV2, perhaps bocaviruses are only "passers-by" in intestinal tract than pathogens of gastroenteritis, or the control participants had symptomless infection of bocaviruses. This issue is not clear yet and needs more studies to resolve [7,8,13,14]. All of the partial *NS1 *gene sequences in this study were closer with the PK-2255 strain (from Pakistani children) than other strains. There was a high sequence identity of 98.5-100% between HBoV2 from case and control groups and the phylogenetic tree also confirmed that there was no difference in the phylogenetic characteristics of them, suggesting that a single genetic lineage of HBoV2 is circulating in both the gastroenteritis and healthy children in Lanzhou, China. And it also indicated HBoV2 can cause asymptomatic infection in Children.

Phylogenetic analyses demonstrated that partial *NS1*, *NP1*, and *VP1 *gene sequences of HBoV2 were markedly similar to those of a number of reference strains. This variability was also reported by Kapoor [2], who postulated that it was due to recombination between HBoV2 strains, as has been reported for animal parvoviruses [15]. However, when phylogenetic trees of non-recombinant segments associated with breakpoints estimated by GARD were compared, Shimodaira-Hasegawa test showed the topologies were not significantly different. Considering that these sequences exhibit very high identity (more than 95%), this recombination phenomenon may be mediated by other processes, for example, variation in spatial rate and/or heterotachy. More works are required to elucidate fully the nature and extent of any recombination that occurs between HBoV2 strains.

Arthur *et al*. identified two recombination sites upstream of the *NS1 *and *VP1/2 *genes, using HBoV, HBoV2, and HBoV3 sequence analyses, and hypothesized that HBoV3 may be a hybrid of HBoV and HBoV2 [3]. Our phylogenetic analyses of HBoV, HBoV2, HBoV3 and HBoV4 strains confirmed that HBoV3 may be a hybrid of HBoV and HBoV4. The estimated breakpoint was located at upstream of the *VP1 *gene. Although bootscanning analyses and phylogenetic trees suggested that HBoV3 was a hybrid of HBoV and HBoV4, the parent strain of the *VP1/VP2 *region was still hard to determine. The *VP1/VP2 *region of HBoV3 was as similar to that of HBoV2 as it was to that of HBoV4, suggesting that HBoV3 may be a hybrid of HBoV and the common ancestor of HBoV2 and HBoV4.

According to the ICTVb criteria http://www.ictvdb.org/Ictv/fs_parvo.htm, isolates with non-structural gene homologies of less than 95% are defined as a new species in the *bocavirus *genus. *NS1 *sequence variation amongst HBoV2 clusters was as high as 8%. However, *NS1 *sequence variation between one HBoV2 cluster and HBoV4 was only 6%, suggesting that either more than one HBoV2 cluster exists or that some of these clusters should in fact be regarded as a separate species. The high sequence identity, limited sequence data, and unclear taxonomy rendered the precise phylogenetic relationships among these human bocaviruses difficult to determine.

The data presented here suggested the potential phylogenetic relationships among the known human bocaviruses. However, many issues remain, including the nature and extent of recombination between HBoV2 strains, and the precise evolutionary relationships of the various human bocaviruses. More studies including more samples from different areas and years are needed to address them.

## Conclusions

In summary, our data suggested that HBoV2 had higher prevalence than HBoV and HBoV3 in both case and control groups. A single genetic lineage of HBoV2 is circulating in children with and without gastroenteritis in Lanzhou, China. Recombination between HBoV2 strains may occur and HBoV3 may be a hybrid virus, originating from HBoV and the common ancestor of HBoV2 and HBoV4.

## Competing interests

The authors declare that they have no competing interests.

## Authors' contributions

Conceived and designed the experiments: ZD, YJ. Performed the experiments: WC, ZX, JY. Analyzed the data: JC, WC, CH, MZ, ZD. Contributed reagents/materials/analysis tools: MJ, HL. Wrote the paper: WC, JC, ZD. All authors read and approved the final manuscript.

## Pre-publication history

The pre-publication history for this paper can be accessed here:

http://www.biomedcentral.com/1471-2334/11/50/prepub
